# Effect of supraneural transforaminal epidural steroid injection combined with caudal epidural steroid injection with catheter in chronic radicular pain management: Double blinded randomized controlled trial.

**DOI:** 10.12688/f1000research.23188.2

**Published:** 2020-07-24

**Authors:** Sithapan Munjupong, Wipoo Kumnerddee

**Affiliations:** 1 Department of Anaesthesiology, Phramongkutklao Hospital and College of Medicine, Bangkok, Bangkok, 10400, Thailand; 2 Department of Rehabilitation Medicine, Phramongkutklao Hospital and College of Medicine, Bangkok, Bangkok, 10400, Thailand

**Keywords:** Transforaminal, caudal, epidural steroid injections, lumbosacral radicular pain, randomized controlled trial.

## Abstract

**Background: **Epidural steroid injection (ESI) has been used in managing chronic radicular pain. Regarding various techniques of ESI, the synergistic effect of caudal ESI (CESI) on transforaminal ESI (TFESI) in chronic lumbosacral radicular pain in prospective randomized controlled trial has not been determined. **Methods**:  A total of 54 eligible patients with lumbosacral radicular pain were randomly allocated to undergo TFESI plus CESI (TC group) or TFESI alone (T group).  The effective response to treatment was predefined by at least a 30% reduced verbal numerical rating scale (VNRS) from baseline between group comparison and the functional outcomes as measured by improved Oswestry Disability Index by least 15 points from baseline. All participants were evaluated using a single blinded outcome assessor before the  procedure and at 1, 3 and 6 months after the procedure. P <0.05 was considered as statistically significant. **Results**:  Average VNRS reduced significantly from baseline after receiving procedure at 1, 3 and 6 months in both groups (P-value <0.05). The TC group exhibited more effective and showed significant pain relief compared with the T group at 3 months (P=0.01). However, no statistical difference was observed between sub group analysis in pain relief and insignificant difference between group comparisons of functional outcomes.

**Conclusions**: A treatment combining TFESI and CESI showed significant pain relief over TFESI alone at 3 months. No effect was found concerning functional evaluation.

**Registration: **Thai Clinical Trials Registry ID TCTR20171101002 01/11/2017F

## Introduction

Chronic lumbosacral radicular pain (CLRP) is a common condition in pain and spine centers. Treatment is challenging among patients who do not respond to either medication or physiotherapy and epidural steroid injection (ESI) is one commonly used intervention to alleviate radicular symptoms ^1^. These inhibit the synthesis of prostaglandins, interrupting nociceptive c fibers and reducing edema surrounding the nerve root ^2– 4^.

Different approaches of ESI are available, namely, transforaminal ESI (TFESI), interlaminar ESI (ILESI), and caudal ESI (CESI) ^5– 9^. The effectiveness of these three injected approaches has been shown. Related studies ^10– 12^ have reported that TFESI was more beneficial than CESI regarding pain relief in herniated disc or radicular pain ^13^. However, one recent systematic review and meta-analysis revealed TFESI could be weakly recommended over CESI ^14^. Furthermore, one retrospective study showed adjunctive CESI on TFESI significant relieved more pain than only TFESI ^15^. Unfortunately, a prospective study has not been conducted of the additional effects of combining the epidural steroid approach. Consequently, this study aimed to compare the effectiveness of pain relief and functional outcome between additional CESI to TFESI and TFESI separately in chronic lumbosacral radicular pain in a prospective randomized study and also to investigate possible complications during injection.

## Methods

### Ethical issues

The study comprised a prospective, single center, randomized, double blind, active-controlled parallel group. Permission to conduct this study was granted by the Institutional Review Board of the Royal Thai Army Medical Ethics Committee and registered with the Thai Clinical Trials Registry on 11 November 2017 ( TCTR20171101002).

### Participants

This study was conducted from November 2017 to January 2019. In total, 54 patients who met inclusion criteria were recruited. Patients attending the PMK Pain Treatment Center, Phramongkutklao Hospital were informed by a nurse anesthetist about the study. Patients who indicated an interest then provided written informed consent. The inclusion criteria comprised patients aged 18 to 80 years old with a history of chronic lumbosacral radicular pain (longer than six months) having a diagnosis of either symptomatology or physical examination correlated using magnetic resonance imaging (MRI) and unsatisfactory pain control with either medication or physiotherapy. The exclusion criteria comprised patients presenting significant neurological deficit or cauda equina syndrome, no absolute contraindications to intervention from MRI such as discitis or spinal infection, coagulopathy, psychiatric problem, pregnancy, language barrier or history of allergy to local anesthetics, triamcinolone and contrast media.

### Randomization and blinding

All patients were interviewed and physically examined by only one pain physician. Pain characteristics were documented and randomly allocated in two groups equally using a randomization with block size of four from a computer-generated table and concealed envelope. The random numbers were kept sealed and opened by a nurse anesthetist uninvolved in this study. Those in the TC group received CESI in addition to TFESI, and the T group received only TFESI. All participants and one nurse anesthetist, trained as outcome assessor were blinded to the study group.

### Interventions

The intervention was performed as a day surgery using local anesthesia. The treatment level determined for supraneural transforaminal approach was based on clinical symptoms correlating with MRI. All patients were placed in the prone position, then pulse oximetry and noninvasive blood pressure were monitored. The lower back and buttocks areas were cleaned using sterile fashion technique.

**Figure 1.**
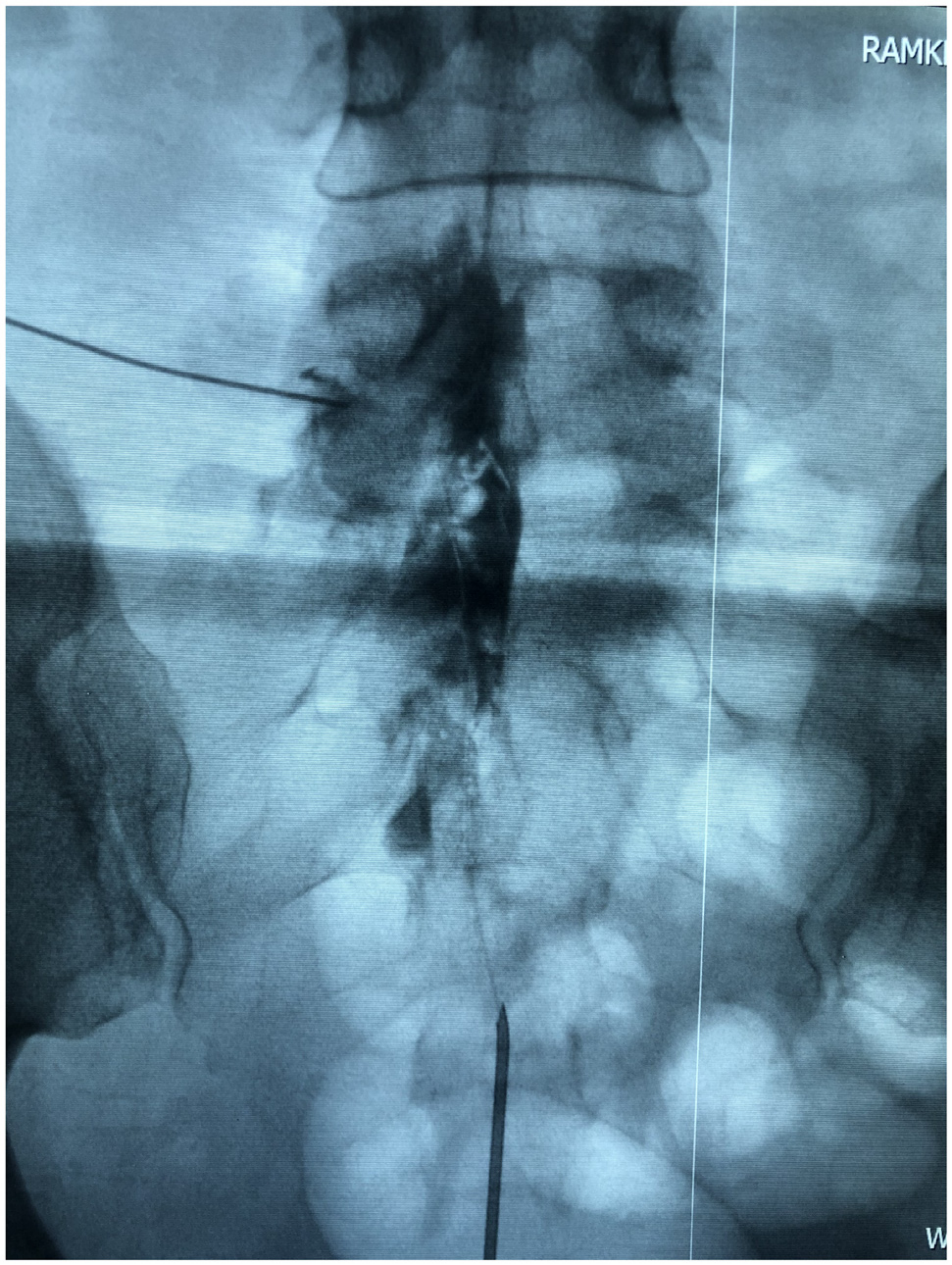
An anteroposterior view of contrast flow in combined supraneural transforaminal and caudal with catheter injection.

**Figure 2.**
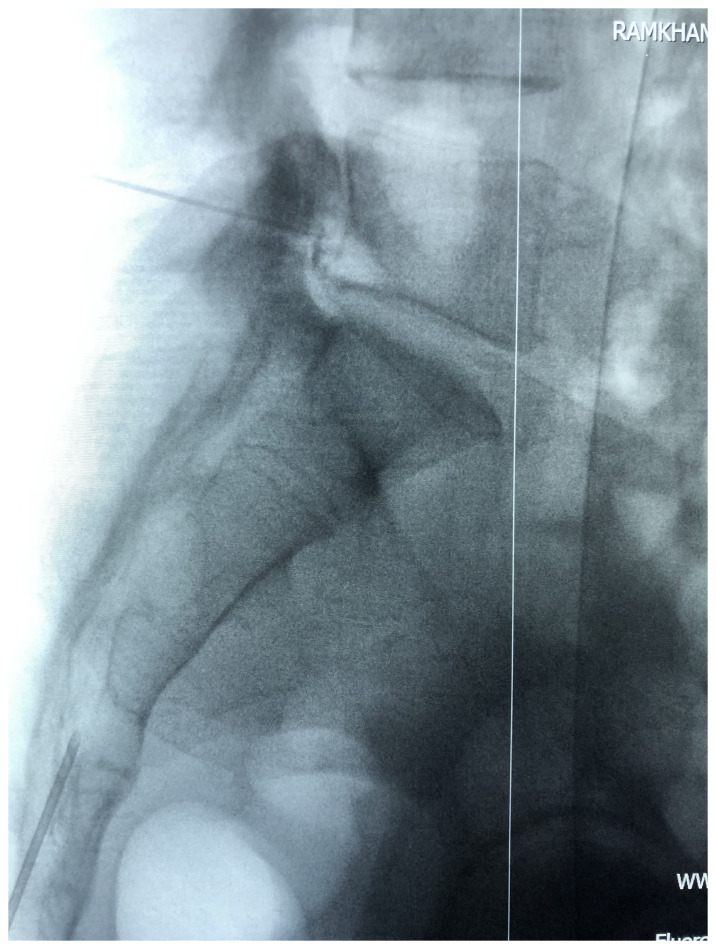
An lateral view of contrast flow in combined supraneural transforaminal and caudal with catheter injection.

A C-arm fluoroscope (9900 Elite, Super C, OEC, UT, USA) was adjusted and rotated obliquely 20 to 25 ^o^ ipsilateral to the affected side and 0 to 10 ^o^ cephalo-caudad tilt until aligned with the superior vertebral end plate. The needle entry points were identified and the skin was infiltrated with 4 to 6 mL 1% lidocaine. A Quinke needle (22-G, 10 cm long) (Unilever, Japan) was inserted in the direction of the radiation beam. The supraneural transforaminal technique was performed, and the tip of the needle was placed below the pedicle and within the upper half of the intervertebral foramen in the lateral image. Then 0.5 to 1 ml of nonionic contrast media (Omipaque 300, GE Healthcare, Shanghai, China) was injected via extension tubing to confirm the needle’s location at the target area under real time fluoroscopy. The caudal epidural space was identified using fluoroscopic guidance in the lateral position and then a 16-gauge introducer Touhy needle (Epimed International RK, Epimed International, Johnstown, NY, USA) was inserted through the sacral hiatus into the caudal space and an epidural catheter was inserted using Touhy needle. Then 0.5 to 1 ml of nonionic contrast media was injected via epidural catheter (Epimed International RK, Epimed International, Johnstown, NY, USA) under real time imaging to confirm the desirable vertebral level and covered target site on both groups as shown in Figure 1 and Figure 2. T-group underwent 0.08% Levobupivacaine (Abbvie S.r.l., Italy) plus 40 mg of triamcinolone (L.B.S. Laboratory Ltd., Bangkok, Thailand) in a total volume of 3 ml via only the intervertebral foramen. Those in the TC group underwent 3 ml of 0.08% Levobupivacaine plus 40 mg of triamcinolone using the transforaminal approach combined with 10 ml of 0.025% Levobupivacaine plus 40 mg of triamcinolone via caudal epidural catheter.

### Outcome measurement

The primary outcome was an effective response to treatment, predefined by at least a 30% reduced verbal numerical rating scale (VNRS; 0-100) from baseline ^16^ between group comparisons. The secondary result was functional outcome, measured by improved Oswestry Disability Index (ODI, Thai version 1) ^17^ at least 15 points from baseline. All participants were completely supervised and evaluated by blinded outcome assessor before the procedure, and then subsequently at 1, 3 and 6 months after procedure when attending the outpatient department of PMK Pain Treatment Center.

### Sample size calculation and statistical analysis

The sample size was calculated based on the related study of Ploumis A *et al.* ^12^. The probability of significantly reduced pain from TFESI was 0.90, whereas the probability of significantly reduced pain from CESI was 0.545. The result indicated 24 patients were required for each group to reach a significance level of 0.05, the power of study was set at 80%, and we added 10% more for loss to follow-up. The final number of participants totaled 27 per group. All analyses and summaries were performed with Stata, Version 13/SE (StataCorp, 2013, College Station, TX, USA). A *P* value <0.05 was considered statistically significant. Descriptive statistics for continuous variables was presented as mean and standard deviation (SD) for sufficiently normally distributed variables. For nominal data, absolute and relative frequencies were displayed for each category. Independent t-test and Chi-square test were performed to compare the differences between groups in continuous variables and categorical variables, respectively. Multilevel mixed linear regression was performed to compare the Verbal Numerical Rating Scale (VNRS) and ODI Questionnaire change over the study period between both groups. A random intercept for the patients was included in the model to account for the cluster structure of the data (two-level models).

## Results

**Figure 3.**
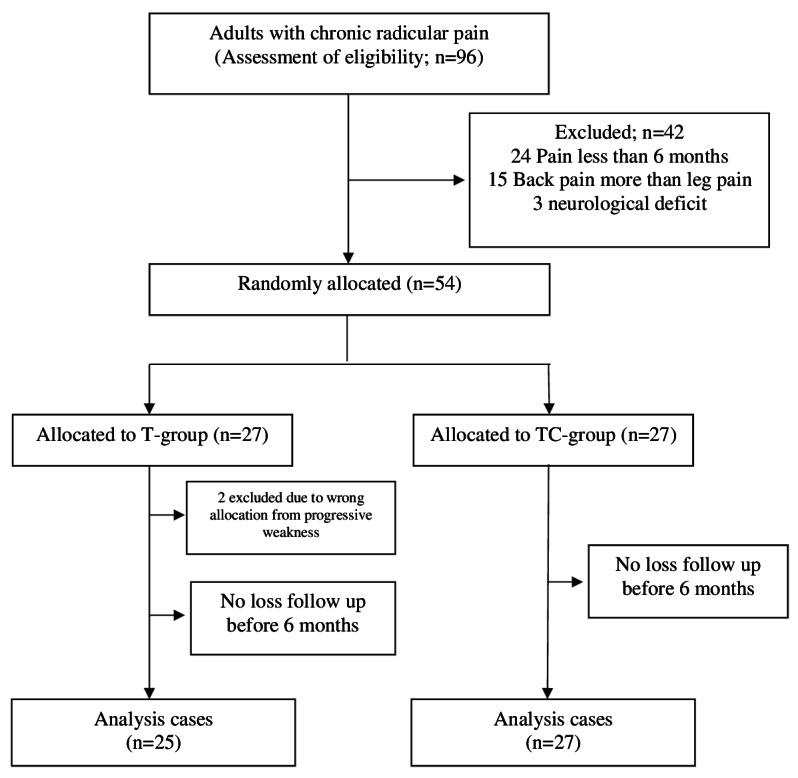
Flow diagram of enrolled patients.

In total, 54 eligible patients were enrolled and allocated in equal groups of 27. Two participants were more excluded due to wrong allocation from progressive weakness and scheduled for surgery in the T group. Consequently, 25 patients remained in the T group and 27 in the TC group as shown in Figure 3. No differences were found regarding demographic data, sex, weight, clinical diagnosis, level of pain dermatome, preprocedural VNRS and ODI before intervention as presented in Table 1 ^18^. Mean baseline VNRS was high in both groups; 74.8 ± 16.8 and 69.6 ± 15.1 in the T and TC groups, respectively.

**Table 1.** Patient characteristics at baseline.

Characteristics	T-group; n=25(%)	TC-group; n=27(%)	p-value
Gender			0.94
Male	16 (64%)	17 (63%)	
Female	9 (36%)	10 (37%)	
Age, mean (SD)	55.4 (15.7)	56.6 (15.9)	0.79
Weight, mean (SD)	67.5 (11.5)	70.1 (11.3)	0.40
Diagnosis			0.99
Disc herniation	6 (24%)	7 (26%)	
Spinal stenosis	7 (28%)	7 (26%)	
Spondylolisthesis	7 (28%)	7 (26%)	
Failed back surgery syndrome	5 (20%)	6 (22%)	
Level of pain dermatome			0.87
L4	1	2	
L5	14	15	
S1	2	1	
L4 and L5	3	5	
L5 and S1	5	4	
VNRS, mean (SD)	74.8 (16.9)	69.6 (15.1)	0.25
ODI, mean (SD)	49.2 (23.3)	44.8 (18.0)	0.44
0% - 20%	3 (12%)	0 (0%)	
21% - 40%	8 (32%)	15 (56%)	
41% - 60%	4 (16%)	6 (22%)	
61% - 80%	8 (32%)	5 (19%)	
81% - 100%	2 (8%)	1 (4%)	

**Figure 4.**
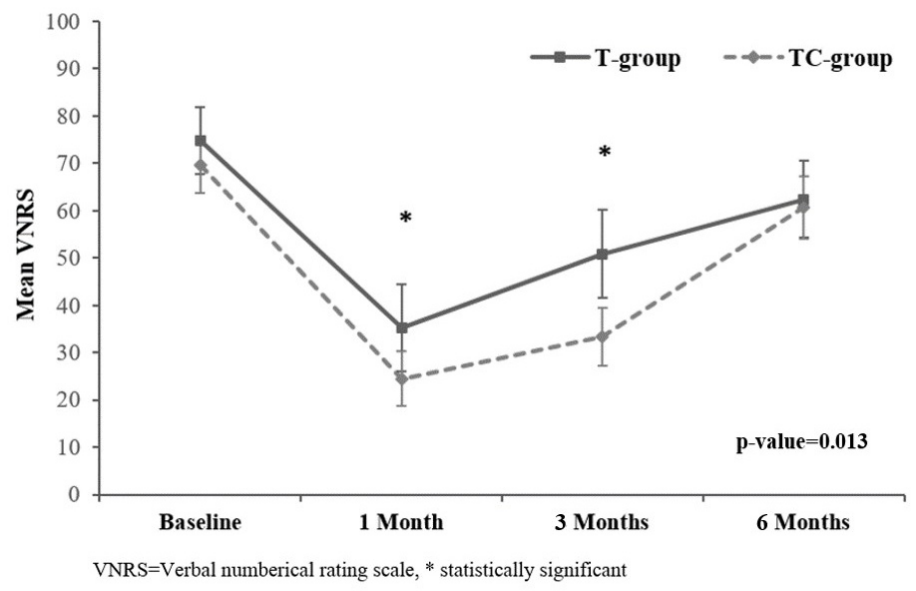
Average verbal numerical rating scale: between-group comparison (Line graph is presented with mean and 95%CI error bars) T group = transforaminal, TC group = transforaminal and caudal.

**Figure 5.**
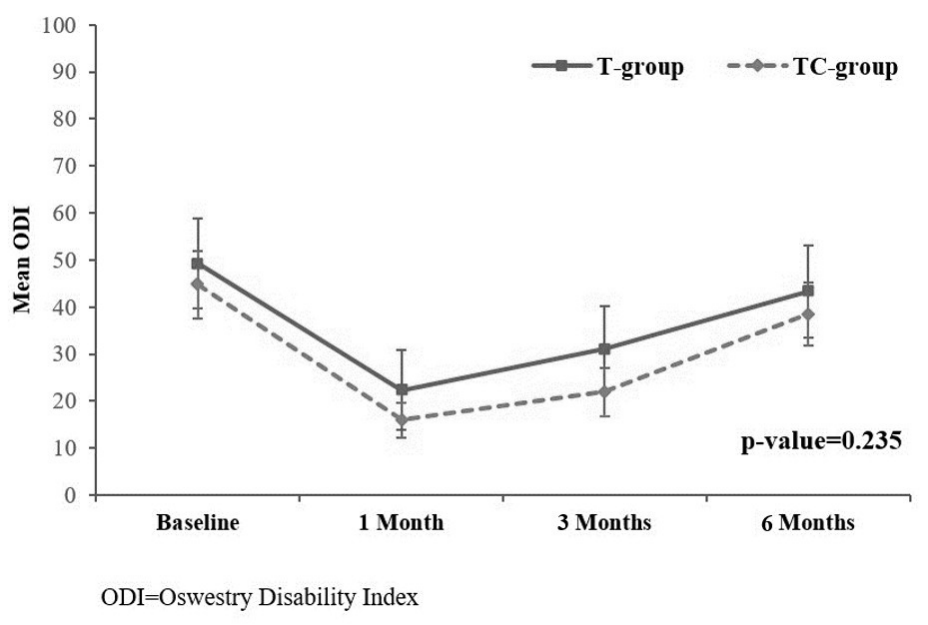
Average Oswestry Low Back Pain Disability Index: between-group comparison (Line graph is presented with mean and 95%CI error bars) T group = transforaminal, TC group = transforaminal and caudal.

Overall, average VNRS was significantly reduced from baseline after receiving the procedure at 1, 3 and 6 months in both groups (P-value <0.05). However, the study showed significant differences between group comparisons at 1 and 3 months (P-value=0.009 and 0.044), respectively) as shown in Figure 4 ^18^. Moreover, average ODI was significantly improved from baseline at 1, 3 and 6 months in both groups. Nonetheless, no significant difference was found in average ODI over the study period between group comparisons (p=0.235) as presented in Figure 5 ^18^.

### Primary outcomes

The number of patients, responding to treatment, was measured by decrease in VNRS of 30% or greater from baseline at each follow-up period, as presented in Table 2 ^18^. This study showed the TC group exhibited more patient responses to the procedure and a significant difference than T group at 3 months follow up (p=0.01). However, there was no significant difference between group comparison in subgroup allocation (p>0.05), as shown in Table 3 ^18^ . Interestingly, the TC group were more satisfied with the treatment outcome in spondylolisthesis and failed back surgery syndrome at 3 months, although not statistical significance (p=0.07 and p=0.08, respectively).

**Table 2.** The number of patients with a decrease in VNRS of 30% or greater from baseline at each follow-up period in chronic lumbosacral radicular pain.

	TFESI (n=25)	TFESI+Caudal (n=27)	p-value
**1 month**	20 (80.0%)	25 (92.6%)	**0.184**
**3 months**	13 (52.0%)	23 (85.2%)	**0.010**
**6 months**	7 (28.0%)	7 (25.9%)	**0.866**

VNRS=Verbal numerical rating scale, TFESI = transforaminal epidural steroid injection, T group = transforaminal, TC group = transforaminal and caudal

**Table 3.** The number of patients with a decrease in VNRS of 30% or greater from baseline at each follow-up period classified by etiologies.

Pain reduction	T-group (n=25)	TC-group (n=27)	p-value
**Disc herniation**			
1 month	6/6	7/7	NA
3 months	4/6	7/7	0.192
6 months	4/6	2/7	0.286
**Spinal stenosis**			
1 month	7/7	6/7	1.000
3 months	5/7	4/7	1.000
6 months	1/7	2/7	1.000
**Spondylolisthesis**			
1 month	5/7	7/7	0.462
3 months	3/7	7/7	0.070
6 months	1/7	2/7	1.000
**Failed back** **surgery syndrome**			
1 month	2/5	5/6	0.242
3 months	1/5	5/6	0.080
6 months	1/5	1/6	0.727

*Statistically significant VNRS=Verbal numerical rating scale, T group = transforaminal, TC group = transforaminal and caudal

### Secondary outcomes

Functional outcome assessed by the number of patients with improved ODI at least 15 points at each follow-up period showed no significant difference between groups, classified by either radicular symptoms or etiology as shown in Table 4 and Table 5 ^18^.

**Table 4.** The number of patients with improvement of Oswestry Disability Index at 15-points or greater at each follow-up period in chronic lumbosacral radicular pain.

	TFESI (n=25)	TFESI+Caudal (n=27)	p-value
**1 month**	16 (64.0%)	21 (77.8%)	**0.273**
**3 months**	12 (48.0%)	18 (66.7%)	**0.173**
**6 months**	7 (28.0%)	10 (37.0%)	**0.488**

TFESI = transforaminal epidural steroid injection

**Table 5.** The number of patients with improvement of Oswestry Disability Index (ODI) at 15-points or greater at each follow-up period classified by etiology.

ODI at least 15 points improvement	T-group (n=25)	TC-group (n=27)	p-value
**Disc herniation**			
1 month	5/6	6/7	0.906
3 months	4/6	5/7	0.853
6 months	4/6	3/7	0.391
**Spinal stenosis**			
1 month	5/7	5/7	1.000
3 months	2/7	2/7	1.000
6 months	0/7	1/7	0.299
**Spondylolisthesis**			
1 month	4/7	6/7	0.237
3 months	4/7	7/7	0.051
6 months	2/7	2/7	1.000
**Failed back surgery** **syndrome**			
1 month	2/5	4/6	0.376
3 months	2/5	4/6	0.376
6 months	1/5	4/6	0.122

T group = transforaminal, TC group = transforaminal and caudal

Additionally, no serious complications, such as neurological deficit, was reported during the course of the study.

## Discussion

This constituted the first prospective study to compare clinical outcomes of combined CESI to TFESI (TC group) and TFESI alone (T group) to alleviate chronic lumbar radicular pain.

This study showed combined CESI with TFESI provided more effective pain relief than TFESI separately at 3 months. Moreover, a trend was shown for higher pain relief in the TC group among spondylolisthesis patients ( total of patients in the TC group compared with 3 of 7 patients in the T group) and fail back surgery syndrome (5 of 6 patients in the TC group compared with 1 of 5 the patients in the T group) at 3 months in subgroup allocation, without significant difference in which prior studies reported the mechanism of radicular pain in spondylolisthesis was usually from mechanical compression resulting in inflammatory changes in the enclosing nerve root and venous and arterial flow disability ^19, 20^. Accessing the epidural space of the supraneural TFESI is relatively difficult in a severely degenerated and narrowed foramen ^13, 21^. Furthermore, the injected volume of lumbar TFESI is likely to influence the results. Prior studies have reported that larger injected epidural volumes provide effective pain relief ^16, 22^ and a larger injected volume can lavage waste products from the epidural space, reducing the abnormal signal of the offending nerve and increasing blood flow to the ischemic nerve ^23^. Desai *et al.* ^24^ confirmed that the more vertebrae covered by the injected volume the better the outcome, and Furman *et al.* ^25^ commented that a larger injected volume was needed for failed back surgery patients. Unsurprisingly, the TC group showed trending toward more pain relief in spondylolisthesis and failed back surgery syndrome.

Unfortunately, this study showed the TC group experienced more significant pain relief than the T group alone at 3 months this might have been from the effect of combined techniques peaking at 3 month, then it might have gradually worn off from steroid’s action, instability and the return of softened epidural adhesion and fibrosis ^15, 22^

However, Friedly JL *et al.* ^26^ found epidural steroid injection plus lidocaine proposed insignificant benefit as compare with lidocaine separately in 400 patients who had lumbar central spinal stenosis and moderate to severe leg pain. On the other hand, they demonstrated interlaminar or transforaminal approaches which were not exactly same techniques as this study.

Recently, a retrospective study reported combined caudal and TFESI in herniated disc provided more significant pain relief and improved patient satisfaction than only TFESI at 1 year ^15^. In contrast, this study showed no significant pain relief between the 2 groups from herniated disc in subgroup allocation, for which demographic data of our patients with herniated disc showed lower average ages (mean age 37 ± 8.5 and 35 ± 5.4 in the T and TC groups, respectively) than a related study (mean age 62.4 ± 15.5 and 57.6 ± 15.7 in the T and TC group, respectively) in which younger patients might have received greater benefit from steroid injection in accordance with Park *et al.* ^27^ showing younger age produced a better response from TFESI. However, no significant difference was observed. Moreover, this study investigated just mild degree herniated disc such as mild unilateral paracentral disc herniation or mild foraminal stenosis and mild degree of spinal stenosis in which only the TFESI technique was sufficient to alleviate pain. Furthurmore, this study injected a larger volume in the T group (3.0 ml) compared with 1.5 ml in the study of TFESI by Kircelli A *et al.* ^15^ in which a larger injected volume could cover more pain generators across multiple levels of the spine in accordance with Furman *et al.* ^25, 28, 29^

In addition, this study postulated that synergistic anti-inflammatory effect from the double dose of steroid in the TC group may have conferred better pain relief. However, one related study reported 40 mg was as effective as 80 mg of methylprednisolone in TFESI for lumbar radicular pain ^29^, while Kang *et al.* ^30^ revealed no significant difference between 10, 20 and 40 mg of triamcinolone at 1 week in TFESI for disc herniation with lumbosacral radicular pain. Unsurprisingly, this study also showed no significant difference in herniated disc concerning different dosages of corticosteroid between the 2 subgroups analysis.

Our study had several limitations. Firstly, we demonstrated radicular pain from symptomatology and physical examination, for which the source of pain may have overlapped the pain referred pattern from the zygophysial joint, sacroiliac joint pain or enclosing soft tissues ^31– 33^, which might have limited the efficacy of the procedure. However, many common problems are involved in chronic low back pain. Secondly, electrodiagnosis was not performed in this study. Nonetheless, electrodiagnosis may have demonstrated false-negative findings, as demonstrated in a similar publication which showed 40 to 85% sensitivity depending on the referral range ^34, 35^. Thirdly, the study did not verify anterior or posterior epidural space of contrast flow which might have affected the efficacy of the result. Fourthly, this study included a small sample size for subgroup allocation which likely underpower analysis and might not have been able to detect differences between subgroups. A larger sample size in each etiology should be demonstrated in further study. Fifthly, this study provided two different approaches, which could be increase costs of procedures. Therefore, the higher volumes should be considerated if the effective result was occurred from higher volumes. Sixthly, some patients had bilateral radicular pain but only one side that more severe pain were provided in T group which might have effected for the result. Seventhly, this study did not collect exactly the duration of pain that can have huge effect on outcome as Bicket *et al*. ^36^ ‘s propose. However, this study included only patients who had history of lumbosacral radicular pain more than 6 months. Lastly, we did not determine whether combination effect of oral medication, physiotherapy and placebo effect which might be effect for the result.

## Conclusion

The additional effect of CESI to TFESI was more effective than TFESI separately at 3 months with no neurological deficit.

## Data availability

### Underlying data

Figshare: data_epidural_04042020.xls. https://doi.org/10.6084/m9.figshare.12320846.v2 ^18^

This project contains the following underlying data:

- data_epidural_04042020.xls (Pain and disability index data for study participants. Data dictionary provided as extended data ^36^)

### Extended data

Figshare: Information of abbreviation data set. https://doi.org/10.6084/m9.figshare.12361499.v1 ^37^

This project contains the following extended data:

- Information of abbreviation data set.docx (Data dictionary)

### Reporting guidelines

Figshare: CONSORT checklist for ‘Effect of supraneural transforaminal epidural steroid injection combined with caudal epidural steroid injection with catheter in chronic radicular pain management: double blinded randomized controlled trial’ https://doi.org/10.6084/m9.figshare.12361490 ^38^
