# Supplementary material for: Effect of supraneural transforaminal epidural steroid injection combined with caudal epidural steroid injection with catheter in chronic radicular pain management: Double blinded randomized controlled trial
Source: F1000Res. 2020 Jul 24;9:634. Originally published 2020 Jun 22. [Version 2] doi: 10.12688/f1000research.23188.2 (PMC7344176; doi:10.12688/f1000research.23188.2)
Supplement: Peer review history

# Peer review: Reviewer response for version 2

**DOI:** 10.5256/f1000research.28023.r67928

**Published:** 2020-08-17

**Authors:** Steven Cohen

**Affiliations:** 1 Department of Anesthesiology, Pain Medicine Division, Johns Hopkins school of medicine, Baltimore, MD, USA

The manuscript seems improved and I'm satisfied with the revision, but I would recommend an English language edit.

Is the work clearly and accurately presented and does it cite the current literature?

Yes

If applicable, is the statistical analysis and its interpretation appropriate?

Yes

Are all the source data underlying the results available to ensure full reproducibility?

Yes

Is the study design appropriate and is the work technically sound?

Partly

Are the conclusions drawn adequately supported by the results?

Yes

Are sufficient details of methods and analysis provided to allow replication by others?

Yes

Reviewer Expertise:

NA

I confirm that I have read this submission and believe that I have an appropriate level of expertise to confirm that it is of an acceptable scientific standard.

# Peer review: Reviewer response for version 2

**DOI:** 10.5256/f1000research.28023.r67927

**Published:** 2020-08-03

**Authors:** Koravee Pasutharnchat

**Affiliations:** 1 Department of Anesthesiology, Faculty of Medicine Ramathibodi Hospital, Mahidol University, Bangkok, Thailand

Thank you the authors for thoughtful responses. The revised article which states 30% or more pain relief as "clinical significant "makes the results more appropriate. Additionally, the authors have added two fluoroscopic imagings which help to determine the deposition of injectate. 

 I appreciate the version 2 of this manuscript, making it more comprehensive and useful.

Is the work clearly and accurately presented and does it cite the current literature?

Yes

If applicable, is the statistical analysis and its interpretation appropriate?

Yes

Are all the source data underlying the results available to ensure full reproducibility?

Yes

Is the study design appropriate and is the work technically sound?

Partly

Are the conclusions drawn adequately supported by the results?

Yes

Are sufficient details of methods and analysis provided to allow replication by others?

Yes

Reviewer Expertise:

Anesthesiology and Pain management

I confirm that I have read this submission and believe that I have an appropriate level of expertise to confirm that it is of an acceptable scientific standard.

# Peer review: Reviewer response for version 2

**DOI:** 10.5256/f1000research.28023.r67926

**Published:** 2020-07-29

**Authors:** Jatuporn Eiamcharoenwit

**Affiliations:** 1 Department of Anesthesiology, Prasat Neurological Institute, Bangkok, Thailand

I agree with your revision. Clinically meaningful pain relief is defined as at least 30% reduction in pain.

Is the work clearly and accurately presented and does it cite the current literature?

Yes

If applicable, is the statistical analysis and its interpretation appropriate?

Yes

Are all the source data underlying the results available to ensure full reproducibility?

Yes

Is the study design appropriate and is the work technically sound?

Yes

Are the conclusions drawn adequately supported by the results?

Yes

Are sufficient details of methods and analysis provided to allow replication by others?

Yes

Reviewer Expertise:

Anesthesiology, Pain management

I confirm that I have read this submission and believe that I have an appropriate level of expertise to confirm that it is of an acceptable scientific standard.

# Peer review: Reviewer response for version 1

**DOI:** 10.5256/f1000research.25596.r65442

**Published:** 2020-07-09

**Authors:** Abdul Hadi Mohamed

**Affiliations:** 1 Department of Anaesthesiology and Critical Care, Kulliyyah of Medicine, International Islamic University Malaysia, Kuantan, Malaysia

**General Comments:**

- This study is very relevant to today's practice and important for patients' wellbeing and continuous healthcare for low back pain with radiculopathy.
- Overall coverages and discussion are appropriate.
- The main limitation for this study is the imaging that showing the flow of contrast which determine of the deposition of steroid as well as the outcome.

**Introduction:**

- Adequate and covering the area of research.

**Methodology:**

- The selection of participants are adequate.
- I am totally agreed with the selection of a common cause of low back pain in this study.

 

**Results:**

- The table and the analysis of the result are adequate.

**Discussion:**

- The coverage of the discussion and suggestion are adequate.
- However, my concern with the selection of participants who is inexperienced without comparing the senior student may affect this study's strength.

**References:**

- Adequate

**Others:**

- This is a good and practical study and should be encouraged to do it multicenter.

This is a very interesting study and can give us options if we may encounter partial or temporary relief with one approach. The limitation of this study is that we can confirm that limited outcome is it due to mechanical that reduces the flow of injected since no contrast was viewed prior to injection.

**Further reading **

1. Rashmi Datta, KK Upadhyay. A Randomized Clinical Trial of Three Different Steroid Agents for Treatment of Low Backache through the Caudal Route. MJAFI 2011; 67: 25-33 ^1^
2. McCormick Z *et al.* Comparison of Pain Score Reduction Using Triamcinolone vs. Betamethasone in Transforaminal Epidural Steroid Injections for Lumbosacral Radicular Pain. Am. J. Phys. Med. Rehabil. 2015 ^2^
3. Tae Kyu Park *et al. *Factors associated with the outcome of transforaminal epidural steroid injections. Korean J Anesthesiol. 2008; 55(3): 298-304. ^3^

Is the work clearly and accurately presented and does it cite the current literature?

Yes

If applicable, is the statistical analysis and its interpretation appropriate?

Yes

Are all the source data underlying the results available to ensure full reproducibility?

Yes

Is the study design appropriate and is the work technically sound?

Yes

Are the conclusions drawn adequately supported by the results?

Yes

Are sufficient details of methods and analysis provided to allow replication by others?

Yes

Reviewer Expertise:

NA

I confirm that I have read this submission and believe that I have an appropriate level of expertise to confirm that it is of an acceptable scientific standard.

## References

[1] : A Randomized Clinical Trial of Three Different Steroid Agents for Treatment of Low Backache through the Caudal Route. *Medical Journal Armed Forces India*.2011;67(1) : 10.1016/S0377-1237(11)80007-9 25-33 10.1016/S0377-1237(11)80007-9 PMC492061427365757

[2] : Comparison of Pain Score Reduction Using Triamcinolone vs. Betamethasone in Transforaminal Epidural Steroid Injections for Lumbosacral Radicular Pain. *Am J Phys Med Rehabil*.2015;94(12) : 10.1097/PHM.0000000000000296 1058-64 10.1097/PHM.0000000000000296 25888660

[3] : Factors Predicting Favorable Short-Term Response to Transforaminal Epidural Steroid Injections for Lumbosacral Radiculopathy. *Medicina (Kaunas)*.2019;55(5) : 10.3390/medicina55050162 10.3390/medicina55050162 31109045PMC6571939

# Response

**Published:** 2020-07-12

**Authors:** Sithapan Munjupong

**Affiliations:** Phramongkutklao College of Medicine, Thailand

Thank you for your review and comment.

 We already edited  and add more contrast flow picture as new version

 Best regards

 Authors

# Peer review: Reviewer response for version 1

**DOI:** 10.5256/f1000research.25596.r65443

**Published:** 2020-07-08

**Authors:** Jatuporn Eiamcharoenwit

**Affiliations:** 1 Department of Anesthesiology, Prasat Neurological Institute, Bangkok, Thailand

1. The primary outcome of this study was an effective response to treatment, predefined by at least a reduced verbal numerical rating scale from baseline. If the cut-off point for the percentage of pain intensity difference was 33%, the primary outcomes may be changed. A 33% pain intensity difference is a standard of the clinically important difference in pain outcome measures. ^1^
2. Figure 2 and figure 3 were alternated figure legends.

Is the work clearly and accurately presented and does it cite the current literature?

Yes

If applicable, is the statistical analysis and its interpretation appropriate?

Yes

Are all the source data underlying the results available to ensure full reproducibility?

Yes

Is the study design appropriate and is the work technically sound?

Yes

Are the conclusions drawn adequately supported by the results?

Yes

Are sufficient details of methods and analysis provided to allow replication by others?

Yes

Reviewer Expertise:

pain management

I confirm that I have read this submission and believe that I have an appropriate level of expertise to confirm that it is of an acceptable scientific standard.

## References

[1] : Defining the clinically important difference in pain outcome measures. *Pain*.2000;88(3) : 10.1016/S0304-3959(00)00339-0 287-294 10.1016/S0304-3959(00)00339-0 11068116

# Response

**Published:** 2020-07-12

**Authors:** Sithapan Munjupong

**Affiliations:** Phramongkutklao College of Medicine, Thailand

Thank you for your review and comment.

 We already edited as new version

 Best regards

 Authors

# Peer review: Reviewer response for version 1

**DOI:** 10.5256/f1000research.25596.r65441

**Published:** 2020-07-06

**Authors:** Koravee Pasutharnchat

**Affiliations:** 1 Department of Anesthesiology, Faculty of Medicine Ramathibodi Hospital, Mahidol University, Bangkok, Thailand

The authors conducted an interesting randomized study comparing transforaminal epidural steroid injection (TFESI) to TFESI combined with caudal ESI. The methodology and flow of the study were organized. The analysis was properly done and results were clear. 

 However, figures 2 and 3 are wrongly placed. The legends of figures state they are average verbal numerical rating scale (VNRS) and average Oswestry Low Back Pain Disability Index (ODI) respectively, while the Y-axes show ODI and VNRS, respectively.

 The discussion is well written. Nevertheless, I think it would be fair if the two groups received the same total dosage of steroid, although many studies did show that two different doses of steroids did not make any differences. Another limitation of such a study, is that the patients were not blinded from their study groups would be a higher placebo effect in the group that received combined injections. 

 Additionally, it would be beneficial if the author had studied comparing cost-effectiveness between these two study groups.

Is the work clearly and accurately presented and does it cite the current literature?

Yes

If applicable, is the statistical analysis and its interpretation appropriate?

Yes

Are all the source data underlying the results available to ensure full reproducibility?

Yes

Is the study design appropriate and is the work technically sound?

Partly

Are the conclusions drawn adequately supported by the results?

Yes

Are sufficient details of methods and analysis provided to allow replication by others?

Yes

Reviewer Expertise:

Anesthesiology and Pain management

I confirm that I have read this submission and believe that I have an appropriate level of expertise to confirm that it is of an acceptable scientific standard.

# Peer review: Reviewer response for version 1

**DOI:** 10.5256/f1000research.25596.r65445

**Published:** 2020-06-24

**Authors:** Steven Cohen

**Affiliations:** 1 Department of Anesthesiology, Pain Medicine Division, Johns Hopkins school of medicine, Baltimore, MD, USA

The authors have performed a small, randomized trial comparing transforaminal epidural steroid injection (TFESI) to TFESI plus add-on caudal ESI. This is an interesting concept, there are not many randomized trials that compare add-on therapy (in essence, a comparative-effectiveness study). I enjoyed reading this and feel it would be a welcome addition to the literature.

**Introduction:**

1. Introduction, para 1: Steroids also suppress ectopic discharges of injured nerves, and may enhance the washout of inflammatory cytokines. This is eloquently noted in the discussion but should be noted at the beginning of the introduction as well.
2. The authors use the term synergistic incorrectly. Synergistic would be 20% + 20% = 50%, but while 2 treatments may afford better results than a single one (see Gilron *et al.* studies for neuropathic medications), the results are usually not even additive.
3. There is a downside to doing a caudal + TFESI even if it works better. There are 2 different billing codes, so it may not be approved. Not only does it increase costs, but it also increases the risk (compared to just using higher volumes or doses).  The authors should, therefore, provide a rationale as to why the combination should work better (the background on the literature is otherwise focused and to the point). 
4. Please consider providing objectives in the ‘introduction’ (the authors do a good job of setting the stage for the study, but specific objectives would be helpful).

**Methods:**

1. I actually like the fact that there was no minimum pain score, as this is always subjective and when a minimum is employed, patients often end up skewed towards the cutoff (i.e. a non-normal curve).
2. On a similar note, I also appreciate that patients with both a herniated disc and spinal stenosis. Were there any differences in outcomes?
3. Many patients with radicular pain have bilateral symptoms. If this were the case, how did the investigators choose what side to inject? Or were these patients excluded (if this is the case, please note it in the ‘methods’)?
4. Why did a nurse anesthetist assess outcomes? Was sedation used (please mention)?
5. Although the authors performed a power analysis, the study is likely underpowered, as well-designed studies now enroll several hundred people (see Friedly *et al.* NEJM, FDA-endorsed CLEAR study) to detect a difference between an ESI and a sham injection, so comparing 2 different ESI should require even more. Yet, the authors have shown a difference.
6. Please consider mentioning the randomization block size.
7. The study had excellent retention (few lost-to-follow-ups), but please note how missing data were handled in the ‘statistical analysis’ section.

**Results:**

1. I agree with one categorical outcome being > 50% pain relief, but the IMMPACT guidelines state that 30% or more relief is ‘clinically meaningful’, so most pain studies now use that cutoff as a positive outcome. According to a review by Bicket MC *et al.* in Pain Pract 2017, an even lower percentage of pain relief seems to predict satisfaction in most patients. So you might consider showing the proportion of people who also obtained > 30% pain relief.
2. Duration of pain is a really important clinical variable that can have a huge effect on outcome (Bicket *et al.* AQUARIUS.. Reg Anesth Pain Med 2018). Please note the duration of pain for the groups.
3. Figures 2 and 3 are wrong because the figure legends states they are showing pain scores & ODI respectively when the y-axes state they are showing ODI and pain scores, respectively (i.e. they’re reversed).

**Discussion: **- in general, well-written and focused.

1. There are 2 possible reasons that warrant mention as to why a difference was shown. One is that the combination group received a higher dose of steroid and local anesthetic (if there had been a group that received a higher dose of steroids by one route as a control that would have formed another control and answered questions). However, there are half a dozen studies that compare 2 different doses of steroids and nearly all have found no difference.
  The second reason is that the group that received both injections had greater expectations, and therefore had a higher placebo response rate. 

Is the work clearly and accurately presented and does it cite the current literature?

Yes

If applicable, is the statistical analysis and its interpretation appropriate?

Yes

Are all the source data underlying the results available to ensure full reproducibility?

Yes

Is the study design appropriate and is the work technically sound?

Partly

Are the conclusions drawn adequately supported by the results?

Yes

Are sufficient details of methods and analysis provided to allow replication by others?

Yes

Reviewer Expertise:

Pain Medicine

I confirm that I have read this submission and believe that I have an appropriate level of expertise to confirm that it is of an acceptable scientific standard, however I have significant reservations, as outlined above.

# Response

**Published:** 2020-07-12

**Authors:** Sithapan Munjupong

**Affiliations:** Phramongkutklao College of Medicine, Thailand

Thank your so much Dr.Cohen for your review and comment.

We already revised the manuscript in Version 2 and have changed the definition of pain relief from 50% to 30%, the conclusion was changed in the subgroup analysis.

All of nurse in our clinic are nurse anaesthetists. Therefore, only one nurse anaesthetist was trained to evaluated the outcome.  However, the patients had not been sedated.

Furthermore, we performed other answers in the revised version.

Best regards

Authors
